# Risk factors of recurrent bacterial vaginosis among women of reproductive age: A cross-sectional study

**DOI:** 10.1515/med-2023-0743

**Published:** 2023-08-08

**Authors:** Xianling Zeng, Ruifang An, Han Li

**Affiliations:** Department of Gynecology, The First Affiliated Hospital of Zhengzhou University, No. 1 East Jianshe Road, Zhengzhou, Henan 450052, China; Department of Obstetrics and Gynecology, The First Affiliated Hospital of Xi’an Jiaotong University, Xi’an 710061, China

## Abstract

The aim of this study was to explore risk factors of recurrent bacterial vaginosis (RBV) among women of reproductive age. This cross-sectional study was carried out in real-world conditions. Women with RBV were selected, and simultaneously uncomplicated bacterial vaginosis (UBV) and those who underwent routine gynecological examination and had normal vaginal microflora were also recruited as the control. Totally, 316 participants were enrolled. Univariate analysis showed that unemployment, desserts, and wiping were related to UBV, while there was no definite relationship between education, high body mass index, smoking, sedentary lifestyle, and RBV or UBV. History of human papillomavirus infection, contraceptive methods, age at first sexual intercourse, and not cleaning vulva during sexual activity were connected with UBV, while the history of other vaginitis and number of sexual partners in the previous year were related to both RBV and UBV. Multivariate logistic regression analysis revealed that lower educational level increased the risk of suffering RBV. Interestingly, no smoking was a protective factor. Moreover, the absence of other vaginitis and an exclusive sexual partner could also weaken the risk of incurring RBV. These various adverse factors alter endocrine function and vaginal immunity, further leading to the recurrence of BV. It is necessary to take corresponding measures to avoid risk factors and to help lessening the prevalence of RBV among women of reproductive age.

## 1 Introduction

Bacterial vaginosis (BV) is the most common vaginal inflammatory disease, which frequently occurs in women of childbearing age. BV is considered to be a disorder of vaginal microbiota characterized by a shift from the *Lactobacillus*-dominant vaginal microbiota to the anaerobes and facultative anaerobes, including *Gardnerella vaginalis*, *Atopobium vaginae*, *Mobiluncus curtisii*, and *Mycoplasma hominis*. Clinically, BV can be diagnosed by satisfying three out of the four characteristics, which include white discharge (thin, homogenous, uniformly adherent), vaginal pH >4.5, fishy odor on addition of 10% potassium hydroxide, and 20% clue cells in vaginal smear microscopy. However, the definition of recurrent bacterial vaginosis (RBV) is still not widely accepted; yet, generally, it is defined as a confirmed diagnosis of BV three or more times within the same year [1].

BV causes thorny public health problems as it increases the risk of health sequelae, especially for childbearing women, their offspring as well as their sexual partners. The adverse concerns include severe reproductive tract infection, abortion, premature birth, premature rupture of membranes, low birth weight infant, neonatal infection, sexually transmitted diseases, cervical lesions, etc. [2,3,4,5]. In addition, BV seriously affects the socioeconomic status and quality of life of patients. What is worse, with the potential to easily cause anxiety and depression, RBV heavily affects women’s work and life [6]. Recent estimates suggest a 30% prevalence of BV among reproductive women globally [7,6]. While variations in prevalence exist among different races and ethnicities. For example, the rate in sub-Saharan Africa may exceed 50% [8].

Unfortunately, although short-term cure rates are generally approximately 80–90% in 3–4 weeks after receiving currently guideline-recommended treatments; yet, more than 50% of women relapse within a year or even within 6 months of treatment. Nevertheless, the possible mechanism of RBV has not been fully demonstrated. Although most BV patients can be effectively treated with antibiotics [9,10]. Yet, recent studies validated that standardized treatment of oral metronidazole has poor efficacy among RBV, showing alarming 76% refractory or recurrent responses [11].

RBV has significant psychosocial impacts on women. It severely affects self-esteem and sex life and carries a high economic burden [6]. Needless to say, RBV is the source of considerably enormous frustration for both practitioners and patients, especially for the latter.

Considering the morbidity of BV and series of economic, psychological, and social burdens caused by BV, it is urgent to explore new strategies to settle this plight. Naturally, etiological prevention is the fundamental approach to decrease the high morbidity and recurrent rate of BV. Therefore, this study aims at screening the risk factors of RBV and UBV among women of reproductive age expecting to offer references for clinical prevention and treatment.

## 2 Materials and methods

### 2.1 Participants

According to the STROBE guidelines, we carried out this cross-sectional study in Gynecology Outpatient Clinic of the First Affiliated Hospital of Xi’an Jiaotong University from June 2016 to June 2019. Inclusion criteria were as follows: (1) women who were 18–50 years old with a history of sexual life; (2) women who had a normal vaginal microflora (NVM) or were diagnosed with uncomplicated bacterial vaginosis (UBV), RBV; and (3) women had a regular menstruation, no vaginal irrigation or drug delivery in the vagina within 7 days, and no sexual intercourse within 3 days before examination. Exclusion criteria were as follows: women who were in the period of pregnancy or lactation and women who had hysterectomy or bilateral salpingo-oophorectomy [12]. Besides, patients with incomplete medical records were also excluded. Then, all women with RBV were included in this present study; at the same time, women with UBV and NVM were also enrolled at a ratio of 1:2. After obtaining each interviewee’s written informed consent, we conducted a face-to-face questionnaire survey of the three groups and analyzed risk factors of RBV. The study protocol was performed with the approval of the ethics committee of the First Affiliated Hospital of Xi’an Jiaotong University.

### 2.2 Diagnostic criteria

BV could be diagnosed using either the Nugent score or the modified Amsel standard regardless of clinical symptoms given the high prevalence of asymptomatic BV. A previous epidemiological study showed that the prevalence of BV was 29% among women at the age of 15–49 when diagnosed by the Nugent score, but only 15.7% of the women had clinical symptoms [13]. While another survey found that the prevalence of BV was as high as 95% by the Nugent score during 2 years of follow-up, but there existed no association between BV and clinical symptoms [14]. In this study, BV was diagnosed by the Nugent score. Vaginal smears were Gram stained and then evaluated under oil immersion (×1,000 magnification) by a single reader and scored using the Nugent scoring criteria (Table 1). Women who had a score of 7 or above were graded as BV. Certainly, a score of 4–6 was graded as intermediate BV, and a Nugent score of 0–3 was graded as an NVM. All the vaginal smears were reevaluated by a second microscopist who was blind to the initial results. If the score differed by more than 2 or resulted in a change in the Nugent category (e.g., from NVM to intermediate BV), then a third reader who was blind to the preceding results was invited to evaluate the smear. At the same time, a meeting was arranged among these three readers to reach a final consensus on any controversial smears. RBV is defined as a confirmed diagnosis of BV three or more times within the same year. Refractory patients were defined as women with persistent BV at the first follow-up visit occurring within 30 days of initiating therapy.

**Table 1:** Nugent scoring criteria

Score	*Lactobacillus* morphotypes	*Gardnerella* and *Bacteroides* spp. morphotypes	Curved Gram variable rods
0	4+	0	0
1	3+	1+	1+ or 2+
2	2+	2+	3+ or 4+
3	1+	3+	
4	0	4+	

0: No morphotypes present, 1+: <1 morphotype present, 2+: 1–4 morphotypes present, 3+: 5–30 morphotypes present, 4+: 30 or more morphotypes present.

### 2.3 Sampling and tests [12]

The patient was in the bladder lithotomic position. One researcher put a speculum lubricated by saline into the vagina, swabbed a bit of vaginal secretion from the posterior fornix by a dry cotton, coated it on the slide from left to right evenly, and put the cotton swab into a clean tube with little saline. Then, the researcher scraped vaginal secretion from the same place by another dry cotton and put the cotton swab into a clean dry tube. The sample was tested by laboratory technicians within 30 min. All the samples were collected by the same researcher and tested by two experienced inspectors at the same time. If the results of the two inspectors were inconsistent, we resorted to a third veteran for the final diagnosis.

### 2.4 Investigation of risk factors

Based on the epidemiological principle, we designed a structured questionnaire survey. Recently, the emerging advancement of molecular and high-throughput sequencing technologies (e.g., next-generation sequencing) has revealed that BV is a multifactorial condition influenced by social, epidemiological, microbiological, and host factors [15]. The survey contains demographic data, such as age, address, profession, education, and body mass index (BMI); living habits, such as eating sweets, desserts, smoking, and physical exercise state; hygienic habits, such as menstrual care, bath way, underwear material, and the forward direction of wiping (forward wiping); previous history, such as history of human papillomavirus (HPV) infection, history of other vaginitis, and reproductive history; and sexual behavior, such as contraceptive methods, the age at first sexual intercourse (FSI), the number of sexual partners in the last year, and frequency of sexual life. Each eligible interviewee was surveyed individually in view of privacy protection by a trained interviewer. Data on the target factors were collected by two researchers separately. Participants were classified by BMI based on the National Institutes of Health and World Health Organization recommendations: underweight (<18.5 kg/m^2^), lean (18.5–24.9 kg/m^2^), overweight (25–29.9 kg/m^2^), and obese (>30 kg/m^2^).

### 2.5 Statistical analysis [12]

All statistical analyses were performed with SPSS version 20.0 and *P* < 0.05 was considered to be of great statistical difference. Univariate analysis was used to calculate crude odds ratios (OR) and 95% confidence intervals (CI). A multiple logistic regression model was used to control confounding factors and to determine which factor still remained statistically significant. Only the factor with the great statistical difference between RBV and BV after univariate analysis was entered into the multivariate logistic regression analysis.

## 3 Results

### 3.1 Demographic characteristics

A total of 316 participants were included in this study under the inclusion and exclusion criteria, including 68 RBV patients, 135 UBV patients, and 113 NVM women. They were at the age of 18–50 and had a regular menstruation. Among women with RBV, 45.58% (31/68) had vulva pruritus, 16.17% (11/68) had vulva discomfort, 27.94 (19/68) manifested yellow leucorrhea, and 7.35% (5/68) showed odor leucorrhea. Among women with UBV, 28.89% (39/135) of them had vulva pruritus, 28.15% (38/135) presented vulva discomfort, 31.11% (42/135) manifested yellow leucorrhea, and 11.85% (16/135) showed odor leucorrhea. While among women with NVM, 10.62% (12/113) of women came to see a doctor for routine gynecological examinations, 62.83% (71/113) had vulva pruritus, 7.96% (9/113) presented vulva discomfort, and 18.58% (21/113) had the symptom of yellow leucorrhea. Majority of the enrolled women (258/316, 81.65%) were less than 45 years old and 58/316 (18.35%) were above the age of 45. There were 126/316 (39.87%) women from rural areas and 190/316 (60.13%) women from urban areas.

### 3.2 Association of RBV with socio-economic factors and hygienic habits

Univariate analysis showed that unemployment, low educational level (secondary or below), high BMI value, frequent smoking, desserts, sedentary lifestyle, and forward wiping were related to BV, no matter RBV or UBV, with great statistical differences (*P*3 < 0.05) (Table 2). Careful screening showed that desserts and wiping were connected with UBV (*P*2 < 0.05) but not RBV. However, occupation, education, BMI, smoking and sedentary lifestyle were associated with RBV, but not with UBV (*P*1 < 0.05).

**Table 2:** Association of RBV with socio-economic factors and hygienic habits

Clinical parameters	RBV	UBV	NVM	*P*1	*P*2	*P*3
	(*n* = 68)	(*n* = 135)	(*n* = 113)			
Age (years)				0.1180	0.4452	0.2736
<30	32	52	52			
30–44	20	60	42			
≥45	16	23	19			
Setting				0.1169	0.9676	0.2319
Rural area	21	57	48			
Town or city	47	78	65			
Occupation				0.0145	0.0013	<0.0001
Unemployment	39	53	23			
Fixed work	29	82	90			
Education				0.0024	0.0011	<0.0001
Secondary or below	47	63	30			
College or above	21	72	83			
BMI (kg/m^2^)				0.062	0.3344	0.014
<18.5	1	11	13			
18.5–24.9	42	92	83			
25–29.9	16	24	12			
˃30	9	8	5			
Smoking				0.0303	0.0374	0.0003
Ever	47	112	104			
Current	21	23	9			
Desserts				0.7898	0.0005	0.0007
Occasionally	41	84	93			
Frequently	27	51	20			
Eating sweets				0.8844	0.8092	0.9698
Occasionally	37	72	62			
Frequently	31	63	51			
Regular exercise				0.0919	0.1619	0.1866
Occasionally or never	22	29	33			
Frequently	46	106	80			
Sedentary lifestyle				0.0364	0.0412	0.0005
Yes	37	52	29			
No	31	83	84			
Measures used during menstruation				0.0663	0.8506	0.1426
Sanitary towel	52	117	97			
Bumf	16	18	16			
Non-menstrual application of pads				0.807	0.1716	0.377
No	45	87	82			
Yes	23	48	31			
Wiping direction after the toilet				0.0856	0.0413	0.0031
Forward	0	43	23			
Backward	38	92	90			
Underwear replacement frequency				0.5014	0.1038	0.1028
>1 day	18	30	16			
≦ 1 day	50	105	97			
Bath way				0.0544	0.8553	0.0798
Showering	51	116	98			
Tubbing	17	19	15			
Underwear material				0.0522	0.6452	0.0548
Pure cotton	46	108	93			
Others	22	27	20			

RBV: recurrent bacterial vaginosis, UBV: uncomplicated bacterial vaginosis, NVM: normal vaginal microflora, BMI: body mass index, OR: odds ratios, CI: confidence interval, *P*1: difference between RBV and UBV, *P*2: difference between UBV and NVM, *P*3: difference among RBV: UBV and NVM, OR: odds ratios, CI: confidence interval.

### 3.3 Association of RBV with previous history, reproductive history, and sexual behaviors

Univariate analysis indicated that history of other vaginitis, history of HPV infection, nonutility of condoms, age at FSI, number of sexual partners in the last year, and not cleaning vulva before or after sexual life were correlated with BV with great statistical differences (*P*3 < 0.05) (Table 3). Rigorous analysis showed that history of HPV infection, contraceptive methods, age at FSI, and not cleaning vulva before or after sexual life were related to UBV (*P*2 < 0.05) but not RBV, while history of other vaginitis and number of sexual partners in the last year were relevant to RBV but not UBV (*P*1 < 0.05).

**Table 3:** Association of RBV with previous history, reproductive history, and sexual behaviors

Clinical parameters	RBV	UBV	NVM	*P*1	*P*2	*P*3
	(*n* = 68)	(*n* = 135)	(*n* = 113)			
History of other vaginitis				0.0088	0.2007	0.0006
No	40	104	95			
Yes	28	31	18			
History of HPV infection				1	0.0151	0.037
Yes	22	45	22			
No	46	90	91			
Pregnancy history				0.8779	0.4166	0.5635
No	25	48	34			
Yes	43	87	79			
History of cesarean section				0.8325	0.718	0.8221
No	21	47	29			
Yes	12	31	22			
Menstrual cycle				0.8824	0.4452	0.713
Follicular phase	37	75	57			
Luteal phase	31	60	56			
Contraceptive methods				0.8443	0.0244	0.0429
Condom	11	22	37			
IUD	21	34	21			
COC	12	25	16			
Others	24	54	39			
Age at FSI (years）				0.1911	0.0002	<0.0001
≤20	24	35	9			
>20	44	100	104			
Sexual life during menstruation				1	0.6944	0.841
No	59	118	101			
Yes	9	17	12			
Number of sexual partners*				0.0344	0.0428	0.0004
1	46	110	103			
≥2	22	25	10			
Frequency of sexual life				0.1736	0.7927	0.3642
More than twice a week	31	48	42			
Less than once a week	37	87	71			
Cleaning the vulva^#^				0.26	0.0128	0.0032
No	24	37	16			
Yes	44	98	97			

RBV: recurrent bacterial vaginosis, UBV: uncomplicated bacterial vaginosis, NVM: normal vaginal microflora, HPV: human papillomavirus, IUD: intrauterine device, FSI: first sexual intercourse, COC: combined oral contraceptive, *Number of sexual partners in the previous year, ^#^Cleaning the vulva during sexual activity, *P*1: difference between RBV and UBV, *P*2: difference between UBV and NVM, *P*3: difference among RBV: UBV and NVM, OR: odds ratios, CI: confidence interval.

### 3.4 Multivariate logistic regression analysis

Backward stepwise regression was used to assess multivariable effects on RBV. Multivariate logistic regression analysis revealed that lower educational level increased the risk of suffering RBV (OR = 2.842, 95% CI = 1.177–6.859, *P* = 0.020). Interestingly, no smoking was a protective factor (OR = 0.371, 95% CI = 0.152–0.885, *P* = 0.026). In addition, the absence of other vaginitis and an exclusive sexual partner could weaken the risk of incurring RBV. However, there was no statistical difference between non-sedentary lifestyle, BMI, and RBV (Table 4).

**Table 4:** Determinants of RBV in a multivariate multinomial logistic regression analysis

Clinical parameters	*β*	SE	Wald	*P*	OR	95% CI
Education						
Secondary or below	1.044	0.45	5.398	0.020	2.842	1.177–6.859
College or above					1	
Sedentary lifestyle						
No	−0.773	0.438	3.114	0.078	0.462	0.196–1.089
Yes					1	
Smoking						
Ever	−1.003	0.449	4.979	0.026	0.37	0.152–0.885
Current					1	
BMI (kg/m^2^)						
≥25	0.818	0.459	3.177	0.075	2.266	0.922–5.569
<25					1	
History of other vaginitis						
No	−2.191	0.658	11.091	0.001	0.112	0.031–0.406
Yes					1	
Number of sexual partners						
1	−1.417	0.512	7.655	0.006	0.242	0.089–0.661
≥2					1	

RBV: recurrent bacterial vaginosis, BMI: body mass index, OR: odds ratios, CI: confidence interval.

## 4 Discussion

In women of childbearing age, BV is the most common vaginal infection and occurs in up to 70% of women. Globally, the prevalence of BV varies considerably between countries, regions, races, and ethnic groups. Although antibiotics are recommended as the first-line therapy, 20–30% of women have a recurrence of symptoms after completing the first round of treatment [16].

Univariate analysis showed that unemployment and a low educational degree were related to UBV, and particularly, lower educational level was closely connected with RBV (*P*1 = 0.0024). The great statistical difference still exited under multivariate logistic regression analysis (*P* = 0.020). Certain studies have believed that the prevalence of lower reproductive tract infections was higher among people with lower socioeconomic status and lower educational background. An epidemiological study in Brazil also argued that high income was a protective factor for BV [17]. This may be due to women with high socioeconomic status and high educational level having more opportunities to gain knowledge of preventing BV and other vaginal infectious diseases. Therefore, they could avoid the occurrence of BV effectively. However, there was a lack of a clear relationship between BV and patients’ age or setting.

Univariate analysis showed that smoking was connected with RBV (*P*1 = 0.0303), and the great statistical difference still exited under multivariate logistic regression analysis (*P* = 0.026). Our results were in accordance with previous literature stating that there was a higher number of women who were diagnosed with BV and had smoking habits [18]. This finding could be interpreted as that smoking promotes an antiestrogen environment and elevates vaginal amines, which predisposes women to BV [19]. Besides, tobacco is thought to alter the physiology and structure of the flora of the vagina, increasing bacterial virulence. A previous study found that compared with lean women, overweight and obese women had a higher frequency of BV [20]. While our result revealed that BMI was statistically different among women with RBV, UBV, and NVM (*P*3 = 0.014). While this difference faded away under multivariate analysis.

This was the first time to take diet and a sedentary life style into consideration when analyzing the risk factors of BV. Univariate analysis revealed that frequent desserts and a sedentary lifestyle were correlated with BV, and particularly, a sedentary lifestyle had more correlation with RBV (*P*1 = 0.0364). While this great statistical difference disappeared under the multivariate logistic regression analysis (*P* = 0.078). The fact that the anus is close to the vagina anatomically provides much convenience for the migration of gut organisms to the vagina. It is hypothesized that overeating desserts and sweets may disrupt intestinal microflora, leading to the imbalance of vaginal microflora, resulting in becoming the chief culprit of RBV. Moreover, a sedentary lifestyle is not beneficial to enhancing the body’s immunity, leaving the body a weakened protective immunity, recalcitrance to overgrown anaerobes, and the hotbed of RBV.

Besides, this study found no statistical relationship between RBV and the menstruation care, underwear materials, frequency of underwear replacement, and bath way. A longitudinal study in Britain also found no significant difference between BV and underwear material and menstrual pads [21]. However, backward wiping as a protective factor for UBV has never been studied before. The reason may be attributed to the fact that the intestinal microflora on the contaminated toilet paper could pollute the vulva and then disturb the vaginal microflora.

This study hinted that history of other vaginitis and history of HPV infection were risk factors for UBV, consistent with another study showing that women with BV were twice as likely to acquire trichomonal vaginitis compared with women without BV [22]. Especially, when it comes to history of other vaginitis, there existed a significant statistical difference (*P*1 = 0.0088). Reproductive history and menstrual cycle had little effect on UBV, but one study found luteal phase to be a protective factor of BV [17]. Many studies hold that use of intrauterine device (IUD) was a risk factor for BV [23,24]. This is perhaps because the tail of IUD exposed in cervical vagina and vagina provided colonization conditions for anaerobic bacteria. Other studies suggested that the use of contraceptives was a protective factor for BV [25,17]. This may be due to the fact that estrogen increases epithelial glycogen, which can metabolize into lactic acid, with antimicrobial activity against BV-associated bacteria. In view of the small number of women who used contraceptives conventionally, it was unscientific to conduct an analysis between contraceptives and BV here. Also, a study stated that hormonal contraception and condom use were protective against BV [26].

The present study showed that there was no significant relationship between sexual intercourse during menstruation, frequency of sexual life, and RBV. Univariate analysis showed that the age of FSI less than 20 and not cleaning the vulva during sexual activity were related to UBV but not RBV. Although BV is not belonging to sexually transmitted disease, it is really associated with unprotected sexual activity [27]. As this study showed, multiple sexual partners increase the susceptibility to RBV and UBV, which was also manifested in another study [1,26]

Interpretations of findings of this study are to be extrapolated cautiously because of its limitations. The primary drawback was that the sample size was not large enough and the data on smoking, alcohol consumption, number of sexual partners, and sexually transmitted diseases were inadequate. Then, the study population was hospital based and the result might not necessarily be applicable to the whole population directly. Therefore, community-based studies are needed. Additionally, the results were inevitably to subject to recall and social desirability bias because all responses received on the baseline questionnaire were self-reported. Consequently, sexual risk behavior may have been underreported in view of privacy protection. There is also limited generalizability of results since only Chinese women were included in this analysis.

## 5 Conclusions

In conclusion, BV poses a great threat to women’s reproductive health, emotional burden, and economic burden. Risk factors of RBV are various, involving women’s hygienic habits, socioeconomic status, disease history, and other aspects. These adverse factors alter the endocrine function and vaginal immunity, further leading to the recurrence of BV. Therefore, it is necessary to take corresponding measures, such as improving living conditions, popularization healthy hygienic education, cultivating personal hygienic behavior habits, strengthening the consciousness of self-protection and avoiding risk factors, expecting to help to lessen the prevalence of RBV, and then unloading psychosocial and economic burden.
